# Providing Timely, Trustworthy Guidance to Frontline Clinicians during a Pandemic

**DOI:** 10.34133/2021/9854894

**Published:** 2021-10-20

**Authors:** Yngve Falck-Ytter, Rebecca L. Morgan

**Affiliations:** ^1^VA Northeast Ohio Health Care System and Case Western Reserve University, Cleveland, Ohio, USA; ^2^School of Medicine, Case Western Reserve University, Cleveland, Ohio, USA; ^3^Department of Health Research Methods, Evidence and Impact, McMaster University, Hamilton, Ontario, Canada

When the unexpected happens, such as a pandemic caused by a novel virus, governmental agencies, organizations, and experts in the field are rightly concerned that the paucity of available evidence may trigger unsubstantiated or erroneous recommendations. The harms of which can be exacerbated with today's massive spread of misinformation through unregulated social media or other trusted information sites, inevitably influencing frontline clinicians' and healthcare workers' behavior and practices. However, recommendations for clinical practices are essential for the prevention, detection, treatment, and management during a global crisis.

In response to the COVID-19 pandemic, clinical and research experts, government agencies, and specialty societies immediately took action to provide guidance to their constituents on how to manage all aspect of the pandemic. Although well-intentioned, the rapid development and publication of consensus recommendations and expert opinions came at the expense of those affected by the pandemic. Lacking the rigor or foundation of evidence-based methods, these documents were at risk for selective reporting, unclear and ambiguous decision-making, and subjective interpretations of the limited evidence, sometimes driven by political views.

During this period, many academic centers, societies, and organizations published trustworthy recommendations following gold standard methods; however, the challenge then becomes discerning between high-quality and potentially misinformed advice. During a period of such uncertainty, yet at the same time information overload, healthcare workers and the general population are forced to identify the best course of action with the lowest risk of undesirable consequences.

In this edition of the Journal, Zhao et al. extensively reviewed COVID-19 guidelines published during the first year of the pandemic, in 2020. Out of hundreds of publications making recommendations, 92 clinical practice guidelines (CPGs) fulfilled minimal criteria for inclusion by presenting management recommendations for COVID-19. Using a rigorous systematic review methodology, the authors meticulously examined adherence to reporting standards (RIGHT statement) [1], assessed the methodological rigor for guideline development using the AGREE-II instrument [2], and judged the trustworthiness by examining whether or not the criteria of the U.S. Academy of Sciences (formerly known as Institute of Medicine or IOM) were successfully applied [3].

The quality of CPG development ratings is expected to be somewhat lower initially in a pandemic when organizations try to rapidly develop recommendations. This is because certain elements may be less feasible in a short time frame, such as consumer or patient involvement or extensive public comment. The authors found this to be true with an initial overall rating percentage in the low 30s across assessment domains. While this rating reflected limitations to the aforementioned areas, it is of concern that even basic elements such as adequate financial conflict of interest (COI) management appeared to be lacking in more than three-quarters of examined CPGs. More than 70% of CPGs did not assess the quality of the underlying evidence nor did they provide a rating of the strength of the recommendation. For guidelines to be trustworthy, it is generally accepted that clinicians need to be informed about limitations of the evidence that may impact whether desired consequences actually outweigh any undesirable consequences. It is important to note the authors' finding that the quality of CPGs appeared to improve over time during 2020.

Despite the IOM's criteria from 2011, less than 10% of the examined CPGs used a systematic review process to inform their recommendations [3]. Selective inclusion of studies that support a particular expert view on COVID-19 is one of the major drivers of biased recommendations, risking the introduction of financial and intellectual conflicts [4]. However, comprehensive checklists exist for the organization, planning, and methodologically rigorous development of clinical practice guidelines [5].

It is likely that at least some of the guideline developers believed that because of the time pressure to develop guidance quickly, a traditional development process would have failed. However, resources to specifically support rapid reviews are available that can successfully guide development teams in the necessary steps to develop methodologically rigorous guidance documents in a time of crisis [6].

Not surprisingly, factors associated with an increased quality score included an evidence-based development process, COI management, and using a rating system for the certainty in the evidence and strength of recommendations, such as GRADE framework. GRADE provides international recognition of desired methodological principles that have been carefully developed, transparently documented, and rigorously tested [7]. It is a common misunderstanding that following the GRADE approach for guideline development is time consuming; rather, without efforts to synchronize the evidence review and decision-making steps, the systematic review process could become the bottleneck.

The authors also showed that the timeliness of incorporating evolving evidence (e.g., the use of corticosteroids for severe COVID-19) was variable, and it appeared that some CPGs did not have the mechanism in place to provide timely updates to their guidance when the effectiveness for interventions had been established. The introduction of “living” reviews and guidelines published in a web-friendly format has largely solved the need for continuous updating in response to the availability of the rapidly changing evidence during a pandemic [8].

Furthermore, newly developed web resources (e.g. https://covid19.recmap.org) exist to provide a continuously updated, interactive map of COVID-19 recommendations. COVID19.recmap.org organizes management recommendations by intervention and presents assessments of the rigor of development. These resources, coupled with studies assessing adherence to gold standard guideline development criteria, will reduce barriers to access for frontline clinicians, policy makers, and the public, as well as assist end-users in discerning high-quality recommendations to make well-informed decisions for health interventions. We hope that raising awareness and providing strategies for development and maintenance of trustworthy guidelines in situations of both limited and quickly evolving information will restore the public's confidence in health recommendations far beyond the reach of the pandemic.

*Yngve Falck-Ytter* *Rebecca L. Morgan*
